# How to become a productive academic writer?

**DOI:** 10.36416/1806-3756/e20240345

**Published:** 2024-11-16

**Authors:** Juliana C Ferreira, Cecilia M Patino

**Affiliations:** 1. Methods in Epidemiologic, Clinical, and Operations Research-MECOR-program, American Thoracic Society/Asociación Latinoamericana del Tórax, Montevideo, Uruguay.; 2. Divisão de Pneumologia, Instituto do Coração, Hospital das Clínicas, Faculdade de Medicina, Universidade de São Paulo, São Paulo (SP) Brasil.; 3. Department of Population and Public Health Science, Keck School of Medicine, University of Southern California, Los Angeles (CA) USA.

## PRACTICAL SCENARIO

As two clinical investigators and educators passionate about building research capacity in Latin America, we designed this scientific methodology short article series in 2015. Since then, more than fifty articles have been published in each issue of the Journal.^1^ Many people have asked us how to keep this pace of writing bimonthly research articles over the years. While many factors contributed to it, the most important determinant for the success of this continued series has been discipline. We carefully planned the series, made a schedule, were very committed, and stuck to it.

## WRITING IS HARD

Writing papers and publishing them is a core activity for clinician-scientists. However, as health professionals, we are trained to care for patients primarily and then conduct research if we choose an academic path. Writing skills are outside the top of the list of most training programs, and early investigators are expected to learn by doing. However, writing is hard, especially when you are a beginner. Consequently, many investigators procrastinate and feel guilty for not writing as much as they should. Procrastination can lead to frustration and impact career choices. Therefore, how do we prevent or overcome the barriers to writing manuscripts and becoming productive scientific writers? In his book: “How to write a lot,”^2^ Paul J Silvia discusses barriers to becoming a productive academic writer and how to overcome these barriers.

## BARRIERS TO WRITING

Among the barriers listed by Silvia, the most important one is the misconception that, in order to write a paper, the investigator must find a large block of time to sit and complete the paper almost all at once, known as binge writing. However, binge writing, as it turns out, is less productive than regular, paced, and scheduled writing. Finding time to write-on weekends, long nights, or during the holidays-does not usually work for the busy clinician-scientist. Instead, allocating periods of time to write in your weekly calendar, for example, 1-2 hours a day, just as you schedule meetings, is very useful. However, it is essential that you stick to the plan, respect that allotted time, and use the time exclusively for writing purposes, just as you allot time in your schedule to go to meetings and to teach your classes.

Other common barriers to avoiding writing include focusing on doing the research (collecting data), reading more papers about the subject matter, or running more data analysis. According to Silvia, the best strategy to overcome these barriers is formally alloting writing time into your schedule. Any activity necessary to complete a writing project can be done during that time.

Finally, a common misconception is that one must be inspired to write. This is a dangerous barrier because we do not control inspiration. However, we can control our schedules. So, instead of waiting for inspiration, like a poet, we suggest coming up with concrete writing goals and committing to writing by scheduling writing time on your calendar. Writing a manuscript can be broken down into small and doable tasks, and you can strategically schedule the most tedious ones for those slow Monday mornings when you are less inspired.

## SETTING GOALS AND MONITORING PROGRESS

After you have allocated time in your schedule to write regularly, the next step is setting your writing goals. These goals are initially broad, such as writing a grant proposal, resubmitting a rejected paper, or starting to write a manuscript of a recently finished clinical study. Make a list of your research projects and set priorities from high to low. Projects with deadlines, such as sending a grant proposal for an open call or replying to reviewers on a paper that has not been rejected but needs revision, are typically high priority since missing the deadline has negative consequences. Then, make a detailed list of tasks for each project. For example, writing a grant proposal may include writing all the specific sections of the grant, updating your CV, and collecting letters of support from co-investigators. A list of small tasks that can be completed in one of your scheduled writing slots is shown in Chart 1.

**Chart 1:** Examples of writing tasks that can be completed during scheduled writing time:

Write 200 words within the data collection subheading of the Methods section
Draw Figure 1
Prepare a table with baseline characteristics of participants in the research study
Add references to the manuscript using a reference manager
Read the reviewers’ comments and make a list of changes to be made to the paper
Check article proofs of an accepted manuscript
Prepare and submit the copyright agreement form for a manuscript you co-authored

Make sure you always have your list of writing projects and the specific tasks handy, possibly taped to your office wall, on an app on your phone, or as a digital document you frequently consult. Finally, monitor your progress by tracking your adherence to scheduled writing and completed tasks. Monitoring does not need to be complicated. Crossing out completed tasks on a task list or sticking post-its onto your computer screen is very rewarding and will help you monitor your pace and boost your confidence as you move towards completing projects.

Finally, Silvia suggests that writing with peers (team writing) can be very helpful. Team writing is a framework that promotes supporting and learning from each other, monitoring each other’s progress, and, ideally, having fun writing together. 

## KEY MESSAGES

- Writing is part of your job as a researcher. Allocate time on your weekly schedule to write and stick to it. Avoid binge writing.
- Create a list of writing projects, prioritize, and break each project into small tasks that you can complete during your scheduled writing times.
- Do not wait for inspiration; writing scientific manuscripts requires discipline and regularity. Sit down on your scheduled writing time and complete a writing task. You can start now!
